# RhoA and vigilin are candidates for immunohistochemical markers for epithelioid malignant mesothelioma

**DOI:** 10.1038/s41598-022-20334-0

**Published:** 2022-11-02

**Authors:** Takuya Hiratsuka, Takushi Yamamoto, Akihiko Yoshizawa, Shinya Toyokuni, Tatsuaki Tsuruyama

**Affiliations:** 1 Department of Drug Discovery Medicine, Medical Innovation Center, Kyoto University Graduate School of Medicine, Sakyo-ku-Yoshida-Konoe-cho, Kyoto, 606-8501 Japan; 2 Clinical Laboratory, Osaka-Fu Saiseikai Ibaraki Hospital, 2-1-45 Mitsukeyama, Ibaraki, Osaka 567-0035 Japan; 3 Kyoto Applications Development Center, Analytical and Measuring Instruments Division, Shimadzu Corporation, 1 Nishino-kyo-Kuwabara-cho, Kyoto, 604-8511 Japan; 4 Center for Anatomical, Pathological and Forensic Medical Research, Kyoto University Graduate School of Medicine, Kyoto, 606-8501 Japan; 5 Department of Pathology and Biological Responses, Graduate School of Medicine, Nagoya University, 65 Tsurumai-cho, Showa-ku, Nagoya, 466-8550 Japan; 6 Department of Molecular Biosciences, Radiation Effects Research Foundation, Minami-ku, Hiroshima 732-0915 Japan; 7 Department of Physics, Graduate School of Science, Tohoku University, Aramaki-Aoba 6-3, Sendai, Miyagi 980-8578 Japan; 8 Department of Tumor Research, Kitano Hospital, The Tazuke-Kofukai Medical Institute, Osaka, 530-8480 Japan

**Keywords:** Cancer, Biomarkers, Oncogenesis

## Abstract

Diagnostic markers of malignant mesothelioma (MM) have been extensively investigated. Immunohistochemistry (IHC) markers, such as calretinin, have been used for pathologic diagnosis. However, more diagnostic markers are required to improve the specificity and sensitivity of pathologic diagnosis. This study proposed two proteins as diagnostic markers for epithelioid MM. One is RhoA, an MM mutation-susceptible locus-derived protein, and another is vigilin, a lung small cell carcinoma marker. IHC was performed using 93 MM (epithelioid, 71 cases; sarcomatoid, 13 cases; and biphasic, 9 cases), 64 lung adenocarcinoma (LAC), 60 lung squamous cell carcinoma (LSC), and 14 normal mesothelial (NM) tissues. The majority of epithelioid MM cases were positive for both RhoA and vigilin, whereas both IHCs showed lower stainability in biphasic and sarcomatoid MM. Besides, both IHCs showed significantly higher stainability for RhoA and vigilin in epithelioid MM than in LAC and LSC (*p* < 0.05). Chi-square tests showed that both RhoA and vigilin IHC positive rate in epithelioid MM was not significantly different from that of calretinin (*p* > 0.05). In the differential diagnosis of MM from lung cancer, the accuracy and specificity of RhoA, vigilin, and calretinin staining were almost equivalent. Further, H-score test showed that there was no significant difference between RhoA versus calretinin and vigilin versus calretinin in IHC positivity in epithelioid MM (*p* > 0.05). In conclusion, RhoA and vigilin may be candidates for immunohistochemical markers for epithelioid MM.

## Introduction

Malignant mesothelioma (MM) is a tumour derived from the mesothelial cells in the serosa that cover the body cavity's inner surface. Exposure to asbestos has been epidemiologically proven to be an etiologic factor for the onset of MM^1^. In Japan, asbestos has affected the health of factory workers as well as members of the general public with no occupational exposure history. According to prevalence statistics data from the Ministry of Health, Labour, and Welfare, Japan, the number of asbestos-related deaths in Japan tripled from 500 in 2005 to 1400 in 2014^2^.

The clinical diagnosis of MM requires a comprehensive analysis of the patient’s history of asbestos exposure, image-based diagnosis, and assessment of hyaluronic acid levels in the pleural effusion. However, the diagnosis of tumours using standardised biochemical tests is challenging because tumour markers or MM-specific proteins necessary for pathological diagnosis have not been identified. It is also difficult to distinguish between MM and pleuritis or to make a differential diagnosis of MM from ovarian cancer, lung adenocarcinoma (LAC), sarcomatoid cancer, and pleurisy^3^. The histological diversity of tumour subtypes also makes diagnosis time-consuming^4^.

Novel techniques using next-generation sequencing to analyse the MM genome have shown that MM has relatively few gene mutations, with an average of 24 amino acid mutations (non-synonymous mutations) reported through exosome analysis^5^. However, detailed reports for MM proteins in tissues are limited. This study aimed to examine RhoA, a mutation-susceptible locus-derived protein, and vigilin (high-density lipoprotein-binding protein; HDLBP), an endoplasmic reticulum (ER)-localised protein, as potential diagnostic markers of epithelioid MM.

The five genes with the highest mutation ratios in MM are *CDKN2A* (cyclin-dependent kinase inhibitor 2A)^6^, *BAP1* (BRCA1-associated protein 1)^7^, *NF2* (neurofibromatosis type 2), *TP53*^8^, and *RhoA*^9,10^. A high-risk germline mutation in the *BAP1* gene has been previously reported for MM, with somatic *BAP1* mutations found to be latent in more than 20% of MM patients^11,12^. In addition to *BAP1* loss^13^, *MTAP*(methylthioadenosine phosphorylase) loss help diagnose mesothelioma tumorigenesis^6,9,14,15^. NF2 has also been a target molecule for MM therapy^16^.

First, we noted RhoA as a candidate marker. Second, we noted vigilin protein, an RNA-binding nuclear-cytoplasmic shuttle protein that is localised to the nucleus and the rough ER. This protein plays a vital role in cellular sterol metabolism^17^ and is one of the candidate markers of lung small cell carcinoma^18^. ER stress has been noted as one of the pathogenic factors of MM^19^. Thereafter, we conducted IHC assay for the two proteins.

## Results

### IHC of RhoA

**Figure 1:**
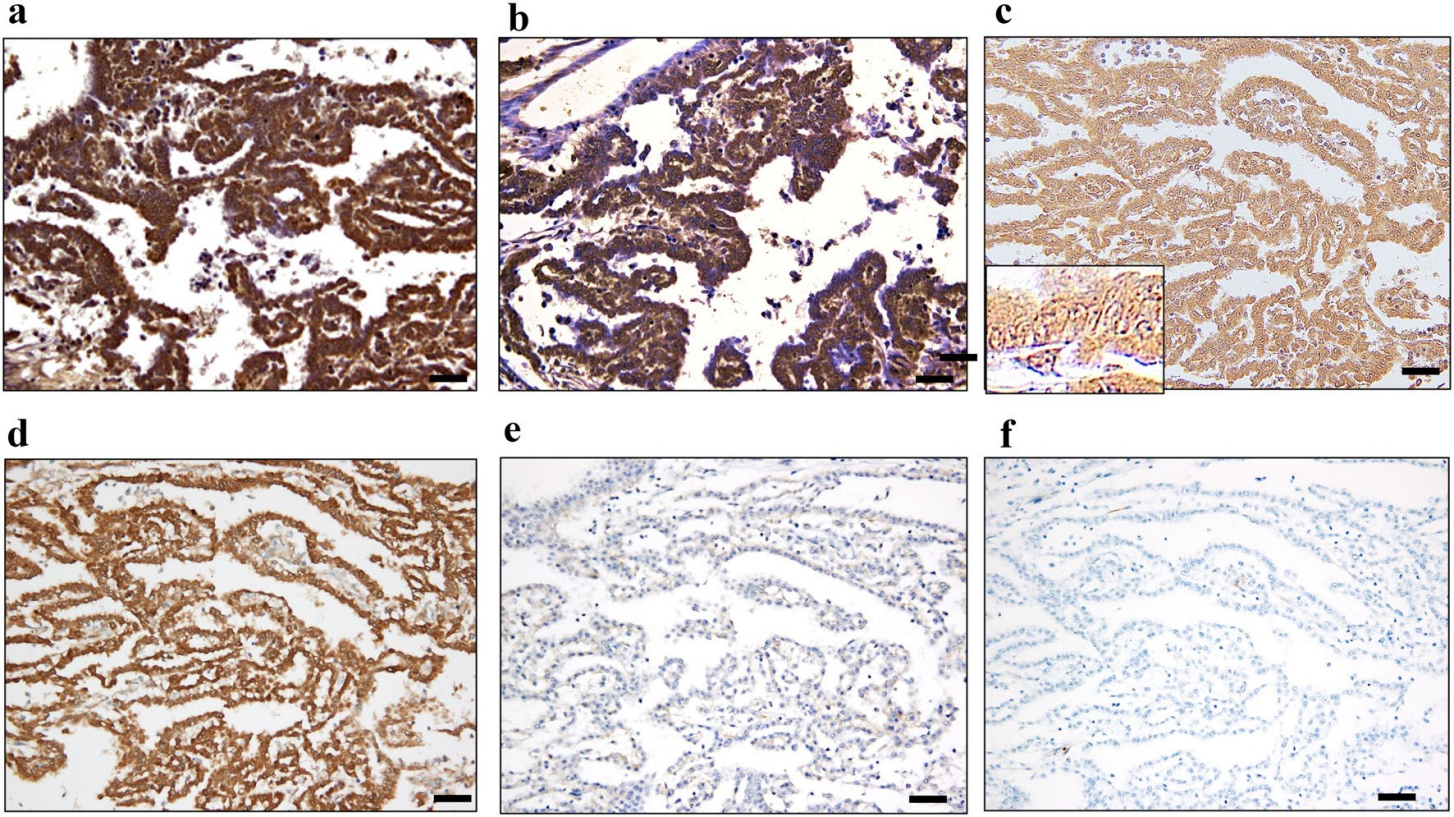
Immunohistochemistry of tissue samples from one representative case of malignant mesothelioma. (**a**) RhoA, (**b**) vigilin, (**c**) D2-40, (**d**) calretinin, (**e**) BAP1, (**f**) WT1. Scale Bars show 50 μm. Original magnification is × 200 for all except the inset photo in (**c**) (× 400).

**Figure 2:**
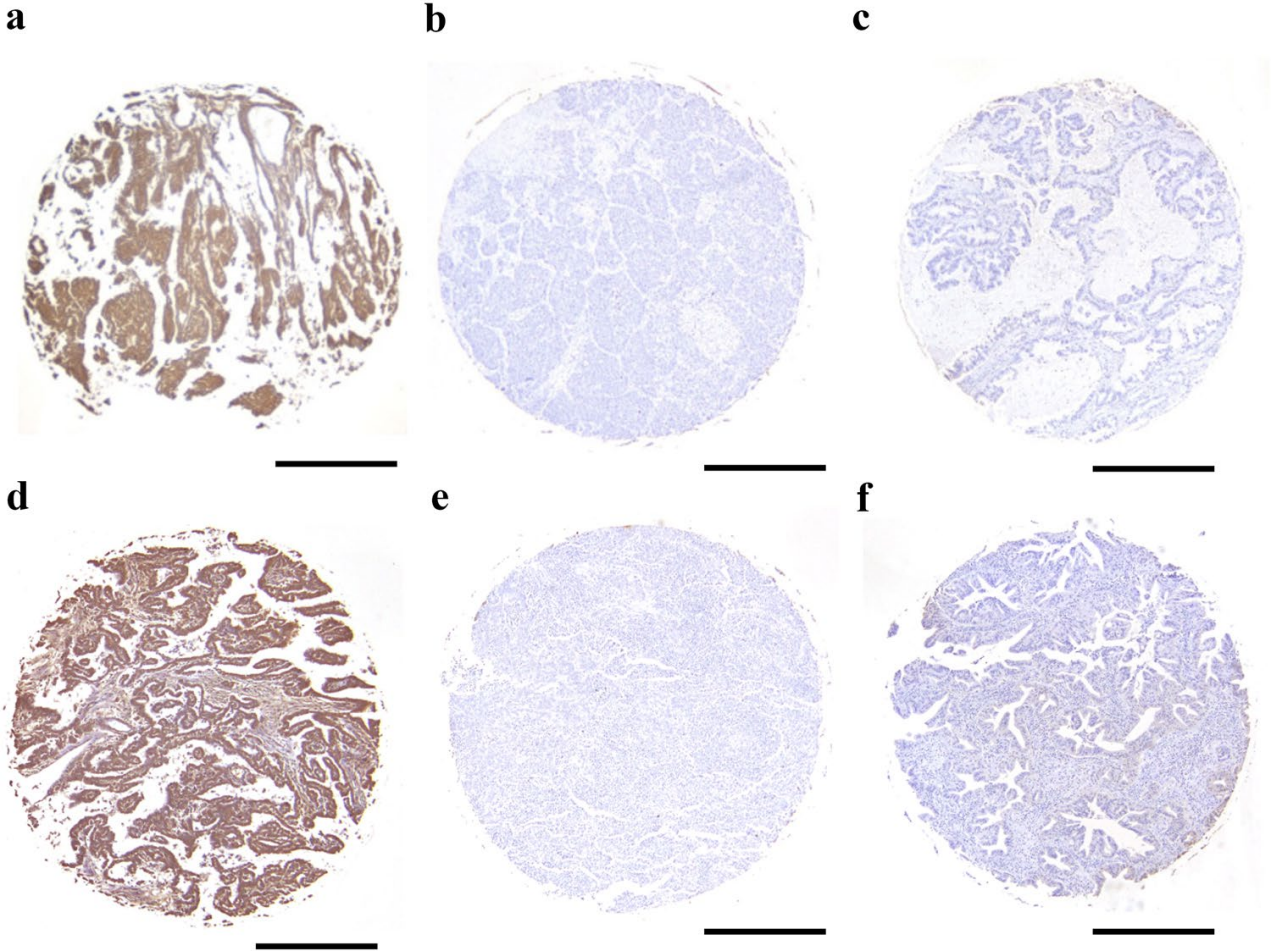
Immunohistochemical image of tissue microarray cores. Diameter, 1.5 mm. (**a**) Epithelioid malignant pleural mesothelioma positive for RhoA; (**b**) Lung squamous cell carcinoma negative for RhoA; (**c**) Lung adenocarcinoma negative for RhoA; (**d**) Epithelioid malignant pleural mesothelioma positive for vigilin; (**e**) Lung squamous cell carcinoma negative for vigilin; and (**f**) Lung adenocarcinoma negative for vigilin. Original magnification is × 100 for all. A scale bar shows 0.5 mm.

Patient characteristics are shown in Table 1. The majority of epithelioid MM cases were positive (overall, 67%; pleural, 88%; abdominal, 50%), and the staining was diffuse in the cytoplasm for RhoA (Fig. 1a and 2a; Tables 2 and 3). Sarcomatoid MM and most of the biphasic MM cases were negative for RhoA (Supplementary Figs. 1a and 2a; Table 2). Normal mesothelial (NM) tissues were also negative for RhoA. RhoA positivity rate in epithelioid MM tissue samples was higher than that in other types of MM, LAC, and LSC tissue samples (7% and 8%), consistent with the results of previous studies^20,21^ (Fig. 2b and 2c; Table 3). Chi-square tests of the RhoA positivity for epithelioid MM showed significant differences in *p*-values compared to that for LAC and LSC (*p* < 0.05) (Table 3). This result suggested that RhoA may be a promising marker for the differential diagnosis of epithelioid MM.

**Table 1:** Patient characteristics.

**Malignant mesothelioma (MM) (*n*)**				
Gender				
Female	47	Male		46
Age				
30 >	8	30‒49		39
50‒69	33	70 <		13
TNM classification (*n*)				
T1N0M0	26	T2N0M0		43
T3N0M0	10	T3N1M0		1
T4N0M0	13			
Histology and anatomic site (*n*)				
	Epithelioid	Sarcomatoid	Biphasic	Total
Pleura				
Pleura	26	10	3	39
Pericardium	7	1	–	8
Subtotal	33	11	3	47
Abdominal cavity				
Mesentery	3	1	2	6
Omentum	6	–	1	7
Peritoneum	29	1	3	33
Subtotal	38	2	6	46
Total	71	13	9	93
**Lung adenocarcinoma (LAC) (*n*)**				
Gender				
Female	24	Male		40
Age				
< 50	21	50 <		43
TNM classification (*n*)				
T2N0M0	38	T2N1M0		9
T2N2M0	1	T3N0M0		6
T3N1M0	2	T2N1M1		1
T4N0M0	1	T4N1M0		4
T4N1M1	1	T4N2M0		1
			Total	64
**Lung squamous cell carcinoma (LSC) (*n*)**				
Gender				
Female	20	Male		40
Age				
< 50	21	50 <		39
TNM classification (*n*)				
T2N0M0	18	T2N1M0		36
T3N0M0	3	T4N1M0		3
			Total	60
**Metastatic lung adenocarcinoma of the peritoneum (*n*)**			2(1 × 2)	
**Normal mesothelial cell (NM) (*n*)**				
Pleural mesothelium			10(5 cases × 2)	
Pericardial mesothelium			18(9 cases × 2)	

Number of patients denoted by *n.*

**Table 2:** Positivity rate of RhoA and vigilin as revealed by immunohistochemistry of malignant mesothelioma (MM).

Anatomic site and MM type	RhoA (%)	Vigilin (%)
**Pleural**		
Epithelioid	23/26 (88)	20/26 (77)
Biphasic	3/10 (30)	0/10 (0)
Sarcomatoid	0/3 (0)	0/3 (0)
**Abdominal cavity**		
Epithelioid	16/32 (50)	26/32 (81)
Biphasic	1/13 (8)	0/13 (0)
Sarcomatoid	1/9 (11)	0/9 (0)
**LAC**	5/64 (7)	5/64 (8)
**LSC**	5/60(8)	3/60 (5)
**Pleural mesothelium**	0/10(0)	0/10(0)
**Pericardial mesothelium**	0/18(0)	0/18(0)

LAC, lung adenocarcinoma, LSC, lung squamous cell carcinoma.

**Table 3:** Immunohistochemistry data for RhoA and vigilin in epithelioid malignant mesothelioma (MM).

RhoA	Positive cases (*n*)	Negative cases (*n*)	Positivity (%)	*p* value*
Epithelioid MM	39	19	67.2	
LAC	5	59	7.2	8.7 × 10^–12^
LSC	5	55	7.7	1.3 × 10^–15^

Vigilin	Positive cases (*n*)	Negative cases (*n*)	Positivity (%)	*p* value*
Epithelioid MM	46	12	79.3	
LAC	5	59	7.8	8.7 × 10^–12^
LSC	3	57	5.0	2.6 × 10^–15^

*p values show the differences in the result of chi-square test between epithelioid MM and lung cancers. LAC, lung adenocarcinoma, LSC, lung squamous cell carcinoma.

### IHC of vigilin

The majority of epithelioid MM cases were positive for vigilin (overall, 79%; pleural, 77%; abdominal, 81%) (Fig. 1b and 2b; Tables 2 and 3). Staining was diffuse in the cytoplasm. Sarcomatoid MM and biphasic MM were negative for vigilin (Supplementary Figs. 1b and 2b). The positivity of LAC and LSC for vigilin was  8% and 5%, respectively (Fig. 2d and 2e; Table 3). Chi-square tests of the vigilin positivity for epithelioid MM showed significant differences in *p*-values compared to that for LAC and LSC (*p* < 0.05) (Table 3). NM cells were negative for vigilin. Parallel to our findings with RhoA, vigilin IHC results clearly distinguish between MM and lung cancer.

### Usability of RhoA and vigilin staining for epithelioid MM diagnosis

**Figure 3:**
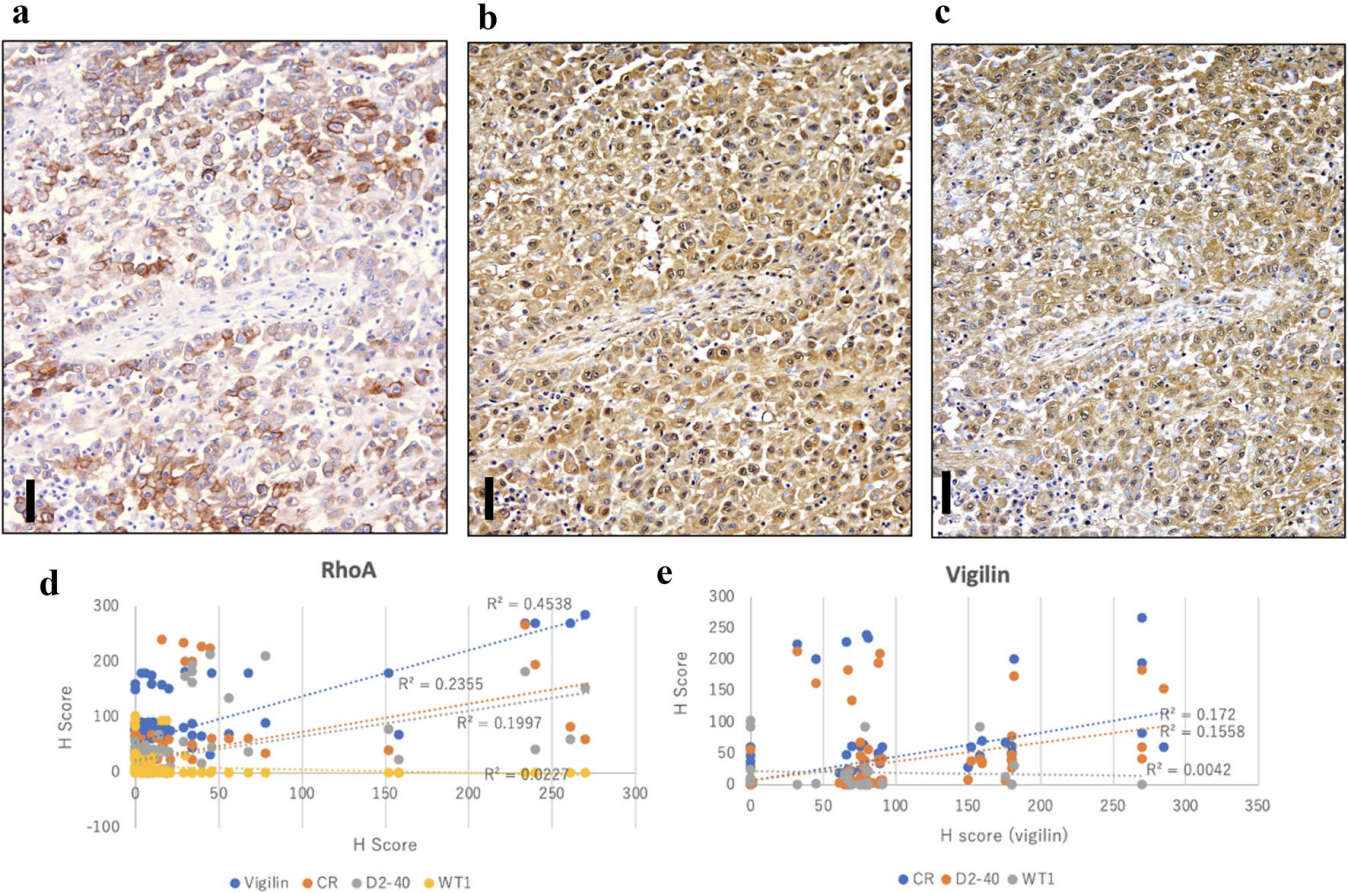
Immunohistochemistry of epithelioid malignant mesothelioma. (**a**) D2-40, (**b**) RhoA, (**c**) vigilin. Scale = 50 µm. Original magnification is 200 × for all. (**d**,**e**) Correlation analysis on H-Score. The horizontal axis represents the H-score of RhoA(left) and vigilin(right). The vertical axis represents each score of  vigilin, calretinin(CR), D2-40, andWT-1. The regression lines are added to the plot graphs. Squares of each correlation coefficient are shown.

We compared the IHC positivity of RhoA, vigilin, D2-40 and calretinin. The results of each immunostaining of epithelioid MM are shown in Table 3 and Fig. 3a–c . The positivity rate for calretinin was 81%, consistent with previously reported results^22^. Besides, The H-score was evaluated based on each IHCstaining intensity and positivity (Fig. 3d and 3e and supplementary Fig. 3a–f). There were no significant differences in the scoring for RhoA (the mean score = 61.0) and vigilin (73.4) compared to that for calretinin (96.7) (*p* = 0.24, Rho A vs. calretinin; *p* = 0.40, vigilin vs. calretinin). The H-score in RhoA IHC showed a moderate correlation with vigilin, D2-40, and calretinin. The H-score in vigilin IHC showed a weak correlation with D2-40 and calretinin.  Finally, chi-square tests of the RhoA and vigilin IHC(positive for epithelioid MM and negativity for lung cancer) showed no significant difference compared to the calretinin IHC (*p* > 0.05) (Table 4). These results showed the same usefulness of RhoA and vigilin in IHC of epithelioid MM as calretinin.

**Table 4:** Immunohistochemistry examination for differential diagnosis of epithelioid MM (positive) from lung cancer (negative).

Epithelioid MM (*n* = 58) versus LC (*n* = 124)	Accuracy	Specificity	Sensitivity
RhoA	0.84	0.86	0.80
Vigilin	0.89	0.91	0.85
Calretinin	0.82	0.90	0.70

Epithelioid MM versus LAC (*n* = 64)	Accuracy	Specificity	Sensitivity
RhoA	0.80	0.76	0.89
Vigilin	0.86	0.83	0.90
Calretinin	0.77	0.79	0.75

Epithelioid MM versus LSC (*n* = 60)	Accuracy	Specificity	Sensitivity
RhoA	0.80	0.74	0.89
Vigilin	0.87	0.83	0.94
Calretinin	0.86	0.82	0.9

LC, lung cancer, LAC, lung adenocarcinoma, LSC, lung squamous cell carcinoma.

## Discussion

This study found that RhoA and vigilin are candidates for novel biomarkers for the diagnosis of MM. RhoA has the GTPase activity and contributes to the cytoskeleton proteins polymerisation. RhoA regulates non-small lung cancer cell migration, but RhoA-related signal cascades in mesothelioma cells are inhibited by crocidolite, a type of asbestos^23^. Crocidolite activates NF-κB and promotes the nitric oxide synthesis by inhibiting the RhoA signalling pathway^24^. Furthermore, RhoA signalling inhibits the migration of breast cancer cells by decreasing stress fibres^25^.

Thus, because RhoA is involved in cell migration, its mutation may affect mesothelioma migration. It is essential to examine the correlation between *RhoA* gene mutations and RhoA protein expression levels. Several mutations are non-synonymous with possible functional damage; however, the expression level is not necessarily reduced^9^. Considering that crocidolite suppresses the RhoA-related signal pathway, the increase in RhoA may be a reactive compensation response. If IHC positivity and the *RhoA* mutation are positively correlated, IHC may be an alternative analytical method. As an IHC marker, RhoA showed less staining variation between tumour cells than did D2-40 in epithelioid MM (Fig. 3a,b). Therefore, RhoA staining is highly stable and useful for the differential diagnosis of epithelioid MM^26,27^.

Vigilin was diffusely positive in the cytoplasm, consistent with its localisation in the ER. We hypothesise that the high IHC stainability of calretinin and vigilin may indicate that ER stress in MM^28^ promotes the accumulation of vigilin^17^. The ER also functions as an intracellular calcium reservoir and is a calcium-mediated signal transmission site. As calcium signalling is tightly regulated by homeostasis, calretinin upregulation may indicate active ER metabolism under asbestos-exposure stress. Also, vigilin may promote hepatocellular carcinoma cell proliferation and tumour growth^29^. Besides, vigilin is downregulated in breast cancer cells and is known to downregulate the post-transcriptional expression of the proto-oncogene *c-fms* encoding CSF-1R in breast cancer^17^. However, it remains unclear whether vigilin is directly involved in the tumorigenesis of epithelioid MM.

The IHC positivity rates of RhoA and vigilin for epithelioid MM were not significantly inferior to that of calretinin^22,30^ (Figs. 2 and 3). Although there was no significant difference in H-score, the IHC stainability of calretinin was more definite than that of both RhoA and vigilin. Therefore, it is necessary to improve the IHC protocols of RhoA and vigilin to obtain more sufficiently stable staining properties in the future for IHC application of both RhoA and vigilin.

As shown in Table 2, the positivity rate of both RhoA and vigilin in biphasic and sarcomatoid MM is not high, suggesting that the pathogenesis of epithelial MM is different from that of sarcomatoid and biphasic MM. A recent study has shown that mesothelial-to-mesenchymal transition (EMT) in mesothelial cells is associated with progression of mesothelioma and its poor prognosis^31^. We demonstrated that both RhoA- and vigilin-positive epithelioid MM cells had retained morphologic features of normal monolayer mesothelial cells (Fig 1a and b). We speculate that mesothelial cells progress to epithelioid MM during EMT, biphasic MM during tumour progression, and sarcomatoid tumours at later stages in parallel with loss of RhoA and vigilin expression. A previous study has demonstrated that the combination of MTAP and BAP1 IHC helps distinguish sarcomatoid mesothelioma from fibrous pleuritis^32^. We will compare RhoA and vigilin positivity with these sarcomatoid markers.

Differentiating between MM cells and LAC cells in pleural fluid is often difficult because of the morphological similarity between these tumours; therefore, immunostaining is helpful. Table 4 shows that the accuracy, sensitivity, and specificity of RhoA, vigilin, and calretinin staining in differentiating lung cancer from epithelioid MM were almost equivalent. For verification of the usability of RhoA and vigilin as diagnostic markers, the sample size was insufficient; however, the pertinence of this study lies in the elucidation of candidates. Furthermore, studies focusing on the comparison between MM, soft tissue tumours, and reactive mesothelial tissues will be necessary for a more accurate differential diagnosis of MM.

In summary, we observed that RhoA and vigilin could serve as reliable immunostaining markers for epithelioid MM. In the future, we hope to confirm and expand on these findings, both with increased numbers of MM cases and by comparing the expressed protein with clinical test data.

## Methods

### Samples

MM, including epithelioid type, normal mesothelial (NM) tissues adjacent to MM lesions, and LAC tissues, obtained as a tissue microarray (mesothelioma: MS801b, MS1001a, MS481d; core size 1.0 mm), LAC (LC641), and lung squamous cell carcinoma (LSC) (BS04041) (US Biomax, Inc., Rockville, MD) were used. Informed consent was obtained from all patients before the collection of primary samples, which were residual tissues, by doctors consulted by Biomax. The study was performed in compliance with the guidelines and with the permission of the Medical Ethics Committee of the Kyoto University Graduate School of Medicine, Japan. No genetic data were obtained. The profiles of 93 MM patients and reference materials, including lung cancer samples (LAC 64 cases, LSC 60 cases, and 28 NM tissues of adjacent tumours) are listed in Table 1. For each patient, two different tissues were used in the analysis to confirm the IHC data. The histology of these tissues was validated by two pathologists using additional IHC of D2-40, WT-1, and calretinin. NM tissues were selected using microscopic analysis. Two pathologists confirmed MM using IHC.

### IHC

RhoA, vigilin, and D2-40 were stained using an EnVision kit (Agilent Life Technologies, Dako, Glostrup, Denmark) following the manufacturer’s protocol. The antibodies used for IHC were RhoA (ab236728, rabbit polyclonal, Abcam, Cambridge, UK), HDLBP/Vigilin (LS‑C440679; LSBio, Seattle, WA), and podoplanin/gp36 (D2-40) (ab77854, mouse monoclonal, clone D2-40, Abcam). All antibodies used were appropriate for IHC of FFPE. The antibodies were diluted 1:20 for staining. To confirm the diagnosis of MM, additional IHC was performed using a Ventana BenchMark AutoStainer (Ventana Medical Systems, Tucson, AZ) with a primary antibody against BAP1 (clone C4, rabbit monoclonal, Santa Cruz Biotechnology, Santa Cruz, CA), WT1 (clone 6F-H2, Mouse, Dako M3561, Agilent, Santa Clara, California), and calretinin (rabbit polyclonal, Invitrogen 08-1211, Waltham, MA). BAP1 staining was considered negative or weak in MM. In IHC for LC641, the cases showing squamous features were excluded because of non-specific staining and tissue damage. We selected TMA cores from 93 cases (2 cores per case for a total of 186 cores) with at least two of these four markers positive and immunostained them with anti-RhoA and anti-vigilin antibodies. The criteria of positivity/retained and negativity in BAP-1 immunostaining was based on a previous study^13^.

### Histological evaluation and chi-square test

H score was determined based on staining intensity and the percentage of stained cells as follows: (% tumour cells-stained 1+ × 1) + (% of tumour cells-stained 2+ × 2) + (% tumour cells stained 3+ × 3)^33^. Scores of 3+, 2+, and 1+ were assigned to each antibody. The following scoring was applied to RhoA, vigilin, and calretinin: 3, definite positivity of > 50% of cells; 2, definite positivity of < 50% of cells or weak positivity of > 50% of cells; 1, weak positivity of < 50% of whole cells; 0, negative staining^34–36^. In case of scores 3 and 2, the IHC for RhoA and vigilin was deemed positive (Supplementary Fig. 3). Chi-square test was performed using Excel software (Redmond, Washington, U.S.).

## Supplementary Information

Supplementary Information 1.

Supplementary Information 2.

Supplementary Information 3.

## Data Availability

The datasets generated and/or analysed during the current study are available from the corresponding author upon reasonable request.

## References

[1] Chew SH, Toyokuni S (2015). Malignant mesothelioma as an oxidative stress-induced cancer: An update. Free Radical Biol. Med..

[2] Nagamatsu Y (2019). Physician requests by patients with malignant pleural mesothelioma in Japan. BMC Cancer.

[3] Muruganandan S (2017). Comparison of outcomes following a cytological or histological diagnosis of malignant mesothelioma. Br. J. Cancer.

[4] Betta PG, Magnani C, Bensi T, Trincheri NF, Orecchia S (2012). Immunohistochemistry and molecular diagnostics of pleural malignant mesothelioma. Arch. Pathol. Lab. Med..

[5] Carbone M, Gaudino G, Yang H (2015). Recent insights emerging from malignant mesothelioma genome sequencing. J. Thorac. Oncol..

[6] Cheng YY (2020). CDKN2A and MTAP are useful biomarkers detectable by droplet digital PCR in malignant pleural mesothelioma: A potential alternative method in diagnosis compared to fluorescence in situ hybridisation. Front. Oncol..

[7] Offin M (2021). Molecular characterization of peritoneal mesotheliomas. J. Thorac. Oncol..

[8] Bueno R (2016). Comprehensive genomic analysis of malignant pleural mesothelioma identifies recurrent mutations, gene fusions and splicing alterations. Nat. Genet..

[9] De Rienzo A (2016). Gender-specific molecular and clinical features underlie malignant pleural mesothelioma. Cancer Res..

[10] Nakamoto M, Teramoto H, Matsumoto S, Igishi T, Shimizu E (2001). K-ras and rho A mutations in malignant pleural effusion. Int. J. Oncol..

[11] Bott M (2011). The nuclear deubiquitinase BAP1 is commonly inactivated by somatic mutations and 3p21.1 losses in malignant pleural mesothelioma. Nat. Genet..

[12] Zauderer MG (2013). Clinical characteristics of patients with malignant pleural mesothelioma harboring somatic BAP1 mutations. J. Thorac. Oncol..

[13] Cantini L (2020). Questioning the prognostic role of BAP-1 immunohistochemistry in malignant pleural mesothelioma: A single center experience with systematic review and meta-analysis. Lung Cancer.

[14] Kinoshita Y (2018). A combination of MTAP and BAP1 immunohistochemistry in pleural effusion cytology for the diagnosis of mesothelioma. Cancer Cytopathol..

[15] Hida T (2017). Immunohistochemical detection of MTAP and BAP1 protein loss for mesothelioma diagnosis: Comparison with 9p21 FISH and BAP1 immunohistochemistry. Lung Cancer.

[16] Ladanyi M (2012). New strategies in pleural mesothelioma: BAP1 and NF2 as novel targets for therapeutic development and risk assessment. Clin. Cancer Res..

[17] Woo HH, Lee SC, Stoffer JB, Rush D, Chambers SK (2019). Phenotype of vigilin expressing breast cancer cells binding to the 69 nt 3'UTR element in CSF-1R mRNA. Transl. Oncol..

[18] Zhou W (2019). A new small cell lung cancer biomarker identified by Cell-SELEX generated aptamers. Exp. Cell Res..

[19] Xu D (2019). Endoplasmic reticulum stress signaling as a therapeutic target in malignant pleural mesothelioma. Cancers (Basel).

[20] Touge H (2007). Diverse activation states of RhoA in human lung cancer cells: Contribution of G protein coupled receptors. Int. J. Oncol..

[21] Gumustekin M (2012). HGF/c-Met overexpressions, but not met mutation, correlates with progression of non-small cell lung cancer. Pathol. Oncol. Res..

[22] King JE, Thatcher N, Pickering CA, Hasleton PS (2006). Sensitivity and specificity of immunohistochemical markers used in the diagnosis of epithelioid mesothelioma: A detailed systematic analysis using published data. Histopathology.

[23] Hu Y (2021). PCGF3 promotes the proliferation and migration of non-small cell lung cancer cells via the PI3K/AKT signaling pathway. Exp. Cell Res..

[24] Aldieri E (2011). Antioxidants prevent the RhoA inhibition evoked by crocidolite asbestos in human mesothelial and mesothelioma cells. Am. J. Respir. Cell Mol. Biol..

[25] Ma L (2015). H2O2 inhibits proliferation and mediates suppression of migration via DLC1/RhoA signaling in cancer cells. Asian Pac. J. Cancer Prev..

[26] Mensi C (2017). Differences between peritoneal and pleural mesothelioma in Lombardy, Italy. Cancer Epidemiol..

[27] Trupiano JK (2004). Diffuse malignant mesothelioma of the peritoneum and pleura, analysis of markers. Mod. Pathol..

[28] Martinotti S, Patrone M, Moccia F, Ranzato E (2019). Targeting calcium signalling in malignant mesothelioma. Cancers (Basel).

[29] Yang WL (2014). Vigilin is overexpressed in hepatocellular carcinoma and is required for HCC cell proliferation and tumor growth. Oncol. Rep..

[30] Ordonez NG (2003). The immunohistochemical diagnosis of mesothelioma: A comparative study of epithelioid mesothelioma and lung adenocarcinoma. Am. J. Surg. Pathol..

[31] Dulong C (2014). The small GTPase RhoA regulates the expression and function of the sodium channel Nav1.5 in breast cancer cells. Int. J. Oncol..

[32] Kinoshita Y (2018). A combination of MTAP and BAP1 immunohistochemistry is effective for distinguishing sarcomatoid mesothelioma from fibrous pleuritis. Lung Cancer.

[33] Thapa B (2016). Calretinin but not caveolin-1 correlates with tumour histology and survival in malignant mesothelioma. Pathology.

[34] Pezzuto F (2020). Prognostic value of Ki67 percentage, WT-1 expression and p16/CDKN2A deletion in diffuse malignant peritoneal mesothelioma: A single-centre cohort study. Diagnostics (Basel).

[35] Fedchenko N, Reifenrath J (2014). Different approaches for interpretation and reporting of immunohistochemistry analysis results in the bone tissue—A review. Diagn. Pathol..

[36] Kim SW, Roh J, Park CS (2016). Immunohistochemistry for pathologists: Protocols, pitfalls, and tips. J. Pathol. Transl. Med..

