# Supplementary material for: RhoA and vigilin are candidates for immunohistochemical markers for epithelioid malignant mesothelioma
Source: Sci Rep. 2022 Nov 2;12:18519. doi: 10.1038/s41598-022-20334-0 (PMC9630375; doi:10.1038/s41598-022-20334-0)

**Supplemental figure 1. Immunohistochemistry of sarcomatoid malignant mesothelioma** (a) RhoA, (b) vigilin, (c) D2-40, d) calretinin, (e) BAP1, (f) WT-1. Scale Bars show 50μm. Original magnification is x200


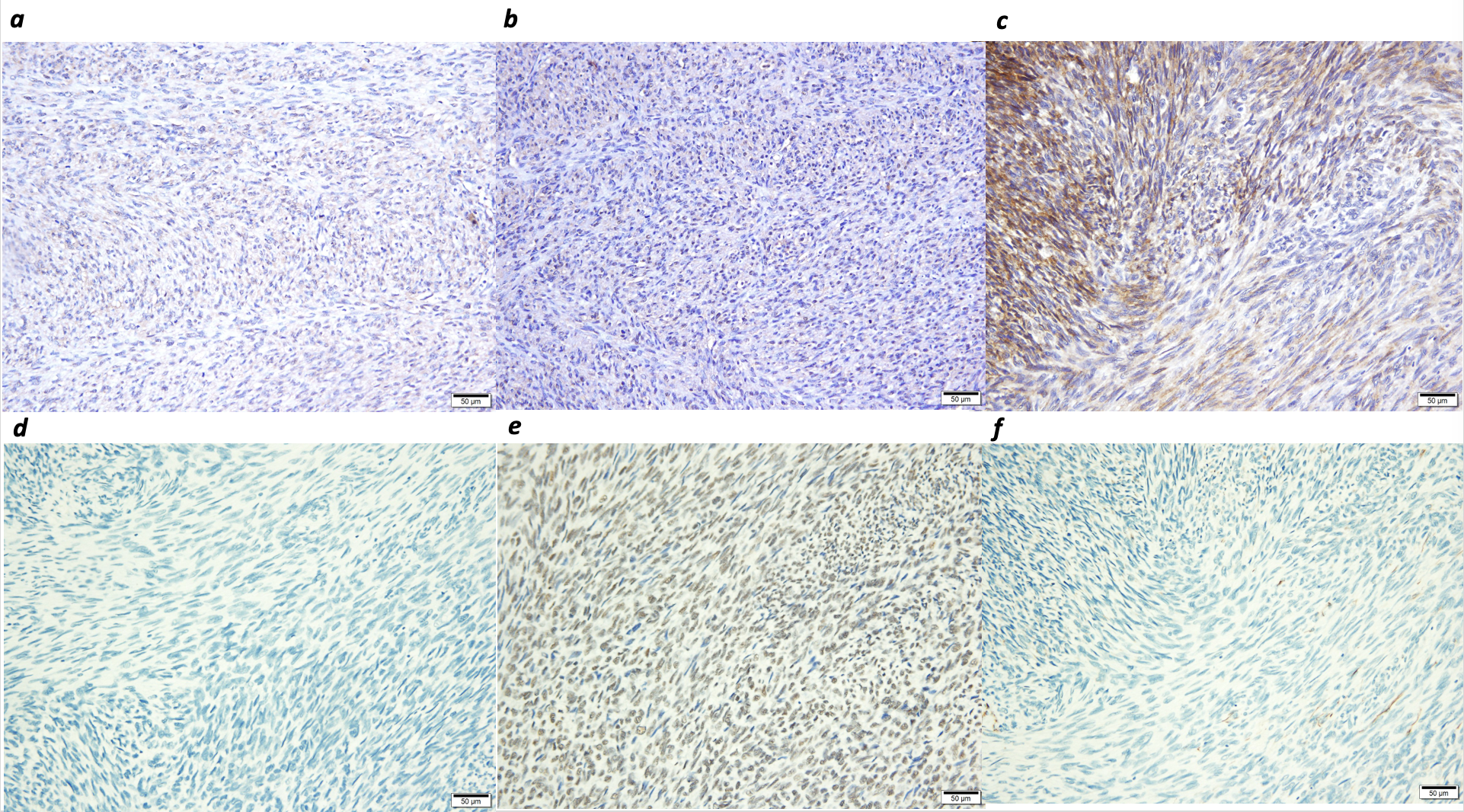

Supplement: Supplementary file 1 — Supplementary Information 1. [file 41598_2022_20334_MOESM1_ESM.docx]
