# Supplementary material for: RhoA and vigilin are candidates for immunohistochemical markers for epithelioid malignant mesothelioma
Source: Sci Rep. 2022 Nov 2;12:18519. doi: 10.1038/s41598-022-20334-0 (PMC9630375; doi:10.1038/s41598-022-20334-0)

**Supplementary figure 3.**

Immunohistochemistry of RhoA and vigilin for scoring: Rho A score 3 (a), score 2 (b), score 1(c), and score 0 (d). Vigilin score 3 (e), score 2 (f), score 1 (g), and score 0 (h). Scale Bars show 50μm. Original magnification is x200.


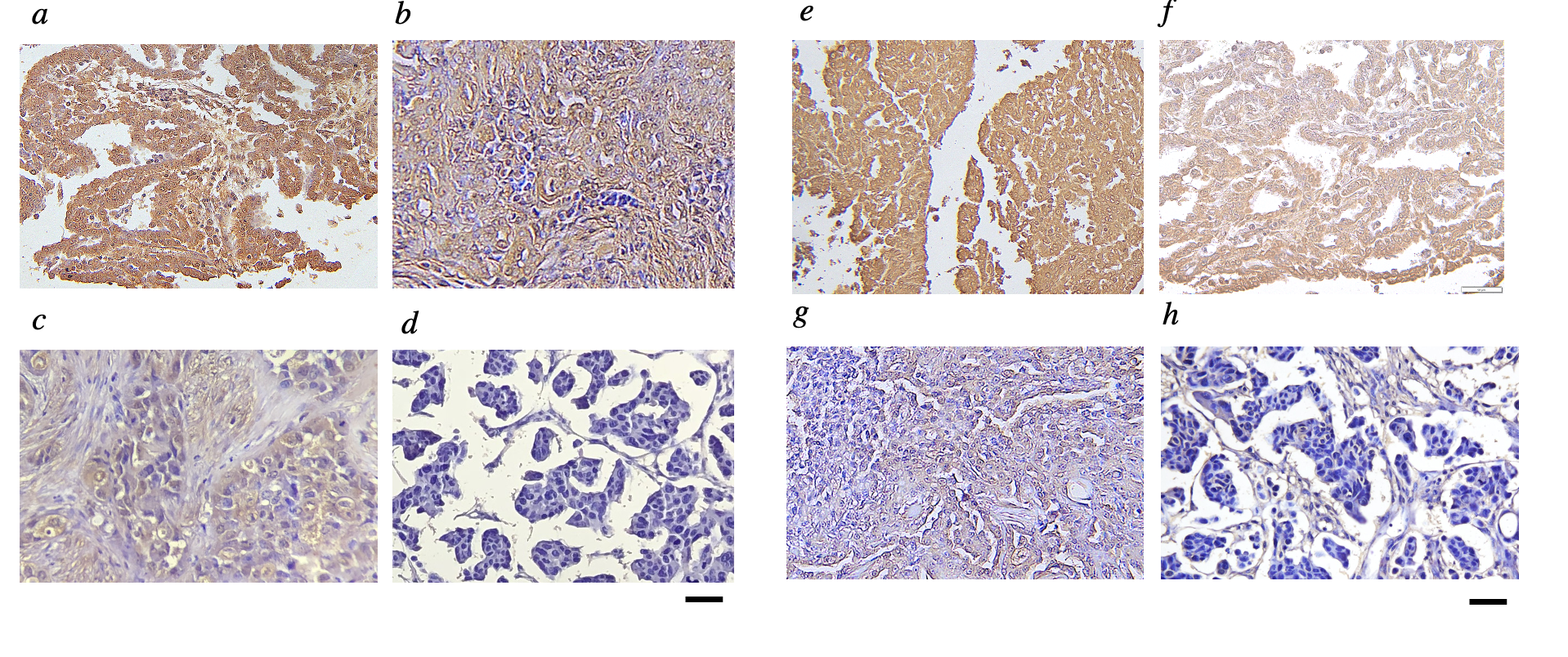

Supplement: Supplementary file 3 — Supplementary Information 3. [file 41598_2022_20334_MOESM3_ESM.docx]
